# Association between fasting plasma glucose and high-sensitivity C-reactive protein: gender differences in a Japanese community-dwelling population

**DOI:** 10.1186/1475-2840-10-51

**Published:** 2011-06-10

**Authors:** Ryuichi Kawamoto, Yasuharu Tabara, Katsuhiko Kohara, Tetsuro Miki, Tomo Kusunoki, Shuzo Takayama, Masanori Abe, Tateaki Katoh, Nobuyuki Ohtsuka

**Affiliations:** 1 Department of Community Medicine, Ehime University, Graduate School of Medicine; Ehime 791-0295, Japan; 2 Geriatric Medicine, Ehime University, Graduate School of Medicine; Ehime 791-0295, Japan; 3 Department of Internal Medicine, Seiyo Municipal Nomura Hospital, Ehime 797-1212, Japan

**Keywords:** C-reactive protein, fasting plasma glucose, type 2 diabetes, gender interaction, risk factor

## Abstract

**Background:**

High sensitivity C-reactive protein (hsCRP) is an acute phase reactant and a sensitive marker of inflammation. Hyperglycemia can potentially promote the production of CRP. The aim of this study was to determine whether increased fasting plasma glucose (FPG) levels are associated with elevated hsCRP concentrations by gender.

**Methods:**

We recruited 822 men (mean age, 61 ± 14 years) and 1,097 women (63 ± 12 years) during their annual health examination from a single community. We cross-sectionally examined whether FPG levels are associated with hsCRP concentrations, and whether this association is independent of gender, body mass index (BMI) and other components of the metabolic syndrome.

**Results:**

In women only, hsCRP increased significantly and progressively with increasing FPG (r = 0.169, P < 0.001). The stepwise multiple linear regression analysis using hsCRP as an objective variable, adjusted for confounding factors as explanatory variables, showed that FPG as well as age, BMI, systolic blood pressure, high-density lipoprotein cholesterol (HDL-C), uric acid, and high molecular weight adiponectin were significantly associated with hsCRP in women, but not in men. There was significant gender interaction, and an increase in hsCRP levels that was greater in women with BMI ≥ 25 kg/m^2 ^and higher FPG than in men.

**Conclusions:**

These results suggested that hsCRP levels increase continuously across the FPG spectrum starting from the lowest FPG in both men and women. However, increase in hsCRP levels was greater in women than men.

## Introduction

C-reactive protein (CRP) is an acute phase reactant and a sensitive marker of inflammation. Several studies support the concept that high-sensitivity C-reactive protein (hsCRP), even when within the clinical normal range, is an important precursor of the metabolic syndrome (MetS) and type 2 diabetes [1-3], and it may be an independent predictor that reflects early stage cardiovascular disease (CVD) [4-6].

A recent review of 20 studies revealed that there was also a significant exponential association between glucose and CVD in nondiabetic participants that extended below the usual "diabetic threshold" [7], and fasting plasma glucose (FPG) is an important predictor of CVD after adjusting for potential confounders. Experimental studies have shown that hyperglycemia stimulates the release of the inflammatory cytokines tumor necrosis factor-alpha (TNF-α) and interleukin-6 (IL-6) from various cells such as monocytes [8]. Recent data have demonstrated that hsCRP is stimulated and produced in the liver by proinflammatory cytokines (e.g., TNF-α and IL-6) produced by visceral adiposity [9]. Thus, elevated FPG is associated with elevated concentrations of hsCRP [10-12].

On the other hand, Nakanishi et al. reported that hsCRP levels are much higher in Westerners than in Japanese [13], and in women compared with men [3,14]. In addition, gender differences have been reported to be consistent across all ethnic subgroups even after multivariable adjustment [14]. However, the question of whether modification by gender has an effect on the association between FPG and inflammation in Japanese has not been investigated in detail.

The aim of this study was to determine whether elevated FPG levels are associated with elevated hsCRP concentrations, and whether this association is independent of gender, body mass index (BMI) and other confounders of CVD. We examined cross-sectional data from Japanese community-dwelling participants.

## Materials and methods

### Subjects

The present study is designed as a part of the Nomura study [15]. Subjects were selected through a community-based annual check-up process in a rural town located in Ehime prefecture, Japan. Information on medical history, present conditions, and drugs were obtained by interview. Other characteristics, such as smoking and alcohol habits, and medication, were investigated by individual interviews using a structured questionnaire. The sample population included 822 men and 1,097 women. The Ethics Committee of Ehime University School of Medicine approved all the procedures and each subject gave informed consent to participate.

### Evaluation of Risk Factors

Information on demographic characteristics and risk factors was collected using clinical files. Body mass index was calculated by dividing weight (in kilograms) by the square of the height (in meters). We measured blood pressure with an appropriate-sized cuff on the right upper arm of the subjects in a sedentary position using an automatic oscillometric blood pressure recorder (BP-103i; Colin, Aichi, Japan) while they were seated after having rested for at least 5 min. Smoking status was classified into never smokers, past smokers, and current smokers. Daily alcohol consumption was measured using the Japanese liquor unit in which a unit corresponds to 22.9 g of ethanol, and the participants were classified into never drinkers, occasional drinkers (<1 unit/day), and daily drinkers (≥1 unit/day). Total cholesterol (T-C), triglycerides (TG), high-density lipoprotein cholesterol (HDL-C), FPG, creatinine (enzymatic method), uric acid, immuno-reactive insulin (IRI), high molecular weight (HMW) adiponectin (Fujirebio, Tokyo, Japan), and hsCRP concentration (Dade Behring Inc., Marburg, Germany) were measured during fasting. Plasma hsCRP concentration was measured using a Behring BN II nephelometer (Dade Behring Inc., Marburg, Germany) and the inter- and intra-assay coefficient variations were 3.2 and 6.7%, respectively. Low-density lipoprotein cholesterol (LDL-C) level was calculated by the Friedewald formula. Participants with TG levels ≥400 mg/dL were excluded.

### Statistical Analysis

Statistical analysis was performed using PASW Statistics 17.0 (Statistical Package for Social Science Japan, Inc., Tokyo, Japan). Data are presented as the mean ± standard deviation (SD) unless otherwise specified, and in cases of parameters with non-normal distributions (TG, FPG, IRI, HMW adiponectin, and hsCRP) the data are shown as median (interquartile range) values. In all analyses, parameters with non-normal distributions were used after log-transformation. Differences among five groups divided according to categories of FPG status and Type 2 diabetes were analyzed by ANOVA or chi-square (*X*^2^) test. Stepwise multiple linear regression analysis was used to evaluate the contribution of each confounding factor for hsCRP. The synergistic effect of gender and the confounding factors was evaluated using a general linear model. ANCOVA was performed using a general linear model approach to determine the association between confounding factors and hsCRP. In these analyses, hsCRP was the dependent variable, the five categories of FPG status were the fixed factors, and confounding factors were added as covariates. A *p*-value < 0.05 was considered significant.

## Results

### Subject background characteristics

The characteristics of the study participants in relation to gender and FPG category are illustrated in Table 1 and Table 2. The study included 822 men, aged 61 ± 14 (range, 20-89) years, and 1,097 women, aged 63 ± 12 (range, 21-88) years. Subjects with type 2 diabetes were the oldest among the men. For both men and women, BMI, systolic blood pressure (SBP), diastolic blood pressure (DBP), prevalence of antihypertensive medication, TG, FPG, IRI, and hsCRP were higher in those with the highest category of normal FPG, IFG, and type 2 diabetes than in those with the lowest and middle categories of normal FPG. History of CVD, HDL-C, and antilipidemic medication in men, and smoking status and HDL-C in women did not differ significantly among the five groups

**Table 1:** Clinical characteristics of male participants according to fasting plasma glucose category

Men, N = 822	Normal fasting plasma glucose			IFG	Type-2 diabetes or >125 mg/dL	*P*-value*
				
Characteristics	FBS <90 N = 200	90-99 N = 286	100-109 N = 143	110-125 N = 63	N = 130	
Age (years)	57 ± 15	60 ± 14	63 ± 13	60 ± 12	66 ± 9	<0.001

Body mass index (kg/m^2^)	22.6 ± 2.9	23.6 ± 2.8	24.5 ± 3.1	24.7 ± 3.2	23.8 ± 2.7	<0.001

Smoking status, %	29.0/19.5/51.5	46.9/22.7/30.4	43.4/29.4/27.3	44.4/28.6/27.0	46.2/30.8/23.1	<0.001

Alcohol consumption, %	15.0/33.0/52.0	12.9/31.8/55.2	17.5/20.3/62.2	3.2/22.2/74.6	20.0/26.2/53.8	0.005

History of CVD, %	7.0	9.1	10.5	9.5	15.4	0.160

Systolic blood pressure (mmHg)	130 ± 17	140 ± 20	147 ± 18	147 ± 19	147 ± 20	<0.001

Diastolic blood pressure (mmHg)	80 ± 10	84 ± 11	87 ± 11	90 ± 12	87 ± 10	<0.001

Antihypertensive medication, %	13.5	22.4	32.9	33.3	33.8	<0.001

Triglycerides (mg/dL)	91 (66-133)	94 (73-126)	104 (75-142)	105 (77-165)	96 (71-162)	0.014

HDL cholesterol (mg/dL)	59 ± 14	59 ± 14	60 ± 16	59 ± 16	58 ± 16	0.880

LDL cholesterol (mg/dL)	105 ± 31	111 ± 29	118 ± 34	107 ± 33	111 ± 33	0.003

Antilipidemic medication, %	2.5	3.5	4.9	1.6	6.9	0.229

Serum uric acid (mg/dL)	5.9 ± 1.2	6.0 ± 1.4	6.1 ± 1.4	6.2 ± 1.4	5.5 ± 1.4	<0.001

Fasting plasma glucose (mg/dL)	86 (83-88)	94 (92-96)	103 (101-105)	115 (112-118)	122 (100--150)	<0.001

Immuno-reactive insulin (*μ*U/mL)	3.65 (2.00-5.48)	4.60 (3.20-6.90)	6.20 (3.50-8.30)	6.60 (4.70-8.70)	4.80 (3.30-8.20)	<0.001

Hypoglycemic medication, %	0	0	0	0	25.4	<0.001

HMW adiponectin (*μ*g/mL)	3.65 (2.37-6.09)	3.41 (1.97-5.52)	3.66 (1.91-5.10)	2.67 (1.70-4.99)	3.03 (1.86-4.88)	0.030

hsCRP (mg/dL)	0.046 (0.026-0.096)	0.046 (0.025-0.104)	0.057 (0.028-0.112)	0.069 (0.042-0.150)	0.060 (0.035-0.117)	0.033

CVD, cardiovascular disease; HDL, high-density lipoprotein; LDL, low-density lipoprotein; HMW. High molecular weight; hsCRP, high sensitivity C-reactive protein. Data presented are mean ± standard deviation. Data for triglycerides, fasting plasma glucose, HMW adiponectin, Immuno-reactive insulin, and hsCRP were skewed, and are presented as median (interquartile range) and were log-transformed for analysis. *P-value: ANOVA or *X*^2 ^-test.

**Table 2:** Clinical characteristics of female participants according to fasting plasma glucose category

Women, N = 1,097 mg/dL	Normal fasting plasma glucose			IFG	Type-2 diabetes or >125 mg/dL	*P *-value*
				
Characteristics	FBS <90 N = 409	90-99 N = 391	100-109 N = 152	110-125 N = 52	N = 93	
Age (years)	58 ± 13	64 ± 10	67 ± 9	68 ± 9	66 ± 10	<0.001

Body mass index (kg/m^2^)	22.5 ± 3.1	23.4 ± 3.2	24.4 ± 3.4	24.9 ± 3.8	24.6 ± 3.9	<0.001

Smoking status, %	97.1/0.7/2.2	98.5/0.5/1.0	95.4/2.0/2.6	98.1/1.9/0	98.9/0/1.1	0.409

Alcohol consumption, %	58.4/35.7/5.9	66.8/26.6/6.6	69.1/27.0/3.9	69.6/28.8/1.9	73.1/24.7/2.2	0.035

History of CVD, %	5.4	5.6	12.5	5.8	9.7	0.025

Systolic blood pressure (mmHg)	131 ± 22	141 ± 22	145 ± 24	151 ± 22	144 ± 23	<0.001

Diastolic blood pressure (mmHg)	77 ± 12	82 ± 11	83 ± 12	86 ± 11	81 ± 11	<0.001

Antihypertensive medication, %	15.6	23.5	38.2	50.0	44.1	<0.001

Triglycerides (mg/dL)	84 (61-114)	92 (69-128)	90 (70-135)	106 (78-144)	94 (70-155)	<0.001

HDL cholesterol (mg/dL)	66 ± 15	65 ± 15	65 ± 16	65 ± 15	61 ± 16	0.127

LDL cholesterol (mg/dL)	120 ± 29	127 ± 29	134 ± 30	131 ± 28	132 ± 31	<0.001

Antilipidemic medication, %	3.7	6.1	9.2	17.3	11.8	<0.001

Serum uric acid (mg/dL)	4.3 ± 1.0	4.5 ± 1.0	4.6 ± 1.1	4.8 ± 0.9	4.6 ± 1.1	<0.001

Fasting plasma glucose (mg/dL)	86 (82-88)	94 (92-97)	103 (101-105)	116 (113-120)	126 (103-150)	<0.001

Immuno-reactive insulin (*μ*U/mL)	4.80 (3.40-6.80)	6.20 (4.50-8.60)	7.40 (5.25-11.5)	9.50 (6.75-12.9)	7.80 (5.20-11.2)	<0.001

Hypoglycemic medication, %	0	0	0	0	37.6	<0.001

HMW adiponectin (*μ*g/mL)	7.15 (4.72-10.3)	6.82 (4.65-10.1)	5.94 (4.12-8.83)	4.82 (3.58-7.73)	5.22 (3.20-8.18)	<0.001

hsCRP (mg/dL)	0.035 (0.017-0.061)	0.043 (0.024-0.089)	0.048 (0.028-0.093)	0.063 (0.031-0.124)	0.080 (0.034-0.198)	<0.001

CVD, cardiovascular disease; HDL, high-density lipoprotein; LDL, low-density lipoprotein; HMW. High molecular weight; hsCRP, high sensitivity C-reactive protein. Data presented are mean ± standard deviation. Data for triglycerides, fasting plasma glucose, immuno-reactive insulin, and HMW adiponectin, and hsCRP were skewed, and are presented as median (interquartile range) and were log-transformed for analysis. *P-value: ANOVA or *X*^2^-test.

### Relationship between hsCRP and various characteristics according to gender

As shown in Table 3, hsCRP increased significantly in correlation with an increase in age, BMI, history of CVD, antihypertensive medication, uric acid, and IRI in both genders, but with a decrease in HDL-C and HMW adiponectin. In addition, hsCRP was also positively correlated with SBP, DBP, TG, LDL-C, antilipidemic medication, FPG, and hypoglycemic medication in women only. IRI was stronger correlated with hsCRP than FPG in both genders.

**Table 3:** Relationship between hsCRP and various characteristics according to gender

	Men, N = 822		Women, N = 1,097	
		
Characteristics	r (*P*-value)	*β *(*P*-value)	r (*P*-value)	*β *(*P*-value)
Age (years)	0.096 (0.006)	0.165 (<0.001)	0.202 (<0.001)	0.211 (<0.001)

Body mass index (kg/m^2^)	0.188 (<0.001)	0.129 (0.002)	0.355 (<0.001)	0.245 (<0.001)

Smoking status, %	0.040 (0.249)	0.102 (0.005)	0.005 (0.863)	--------

Alcohol consumption, %	-0.017 (0.637)	--------	-0.080 (0.008)	--------

History of CVD, %	0.086 (0.013)	--------	0.073 (0.016)	--------

Systolic blood pressure (mmHg)	0.049 (0.164)	--------	0.126 (<0.001)	-0.081 (0.009)

Diastolic blood pressure (mmHg)	0.028 (0.426)	--------	0.103 (0.001)	--------

Antihypertensive medication, %	0.115 (0.001)	--------	0.138 (<0.001)	--------

Triglycerides (mg/dL)	0.040 (0.257)	-0.099 (0.012)	0.209 (<0.001)	--------

HDL cholesterol (mg/dL)	-0.190 (<0.001)	-0.152 (<0.001)	-0.196 (<0.001)	-0.061 (0.040)

LDL cholesterol (mg/dL)	0.028 (0.420)	--------	0.179 (<0.001)	--------

Antilipidemic medication, %	0.036 (0.301)	--------	0.079 (0.009)	--------

Serum uric acid (mg/dL)	0.126 (<0.001)	0.117 (0.001)	0.285 (<0.001)	0.164 (<0.001)

Fasting plasma glucose (mg/dL)	0.067 (0.056)	--------	0.232 (<0.001)	0.121 (<0.001)

Immuno-reactive insulin (*μ*U/mL)	0.162 (<0.001)	0.087 (0.040)	0.263 (<0.001)	--------

Hypoglycemic medication, %	0.027 (0.445)	--------	0.082 (0.007)	--------

HMW adiponectin (*μ*g/mL)	-0.094 (0.007)	--------	-0.197 (<0.001)	-0.107 (0.001)

R^2^	--------	0.090 (<0.001)	--------	0.227 (<0.001)

hsCRP, high sensitivity C-reactive protein; HDL, high-density lipoprotein; LDL, low-density lipoprotein; HMW. High molecular weight. Data presented are mean ± standard deviation. Data for triglycerides, fasting plasma glucose, immuno-reactive insulin, and hsCRP were skewed and log-transformed for analysis. *r, Pearson's correlation coefficient; β, standard regression coefficient.

The stepwise multiple linear regression analysis using hsCRP as an objective variable, adjusted for confounding factors as explanatory variables is shown in Table 3. In men, age, BMI, smoking status, TG, HDL-C, uric acid, and IRI were significantly associated with hsCRP, and in women, age, BMI, SBP, HDL-C, uric acid, FPG, and HMW adiponectin were significantly associated with hsCRP.

### General linear model for interactions between gender and confounding factors

To further investigate whether the interaction between gender and FPG, and the confounding factors could influence hsCRP, a general linear model for hsCRP was analyzed using the parameters in Table 3 and gender including interaction between gender and smoking status, SBP, TG, IRI, FPG, and HWM adiponectin (Table 4). The results revealed that the interaction between gender and FPG (*F *= 5.547, *P *= 0.019) was significantly associated with hsCRP, in addition to gender, age, BMI, SBP, HDL-C, uric acid, FPG, and HMW adiponectin. This finding indicates that the association between FGB and hsCRP was significantly different between men and women.

**Table 4:** Gender interaction between hsCRP and various subject characteristics

	N = 1,919
	
Characteristics	F (*P*-value)
Gender (men = 0, Women = 1)	4.573 (0.033)

Age (years)	68.24 (<0.001)

Body mass index (kg/m^2^)	59.82 (<0.001)

Smoking status	1.925 (0.165)

Systolic blood pressure (mmHg)	4.839 (0.028)

Triglycerides (mg/dL)	1.452 (0.228)

HDL cholesterol (mg/dL)	12.82 (<0.001)

Serum uric acid (mg/dL)	38.38 (<0.001)

Fasting plasma glucose (mg/dL)	13.30 (<0.001)

Immuno-reactive insulin (*μ*U/mL)	1.491 (0.222)

HMW adiponectin (*μ*g/mL)	10.27 (0.001)

Gender *Smoking status	0.751 (0.386)

Gender *Systolic blood pressure	0.050 (0.823)

Gender *Triglycerides	0.845 (0.358)

Gender *Immuno-reactive insulin	0.056 (0.814)

Gender *Fasting plasma glucose	5.547 (0.019)

Gender *HMW adiponectin	1.709 (0.191)

hsCRP, high sensitivity C-reactive protein; HMW, high molecular weight. Data for hsCRP, triglycerides, fasting plasma glucose, immuno-reactive insulin, and HMW adiponectin were skewed and log-transformed for analysis.

### Relationship between FPG and hsCRP according to gender

**Figure 1:**
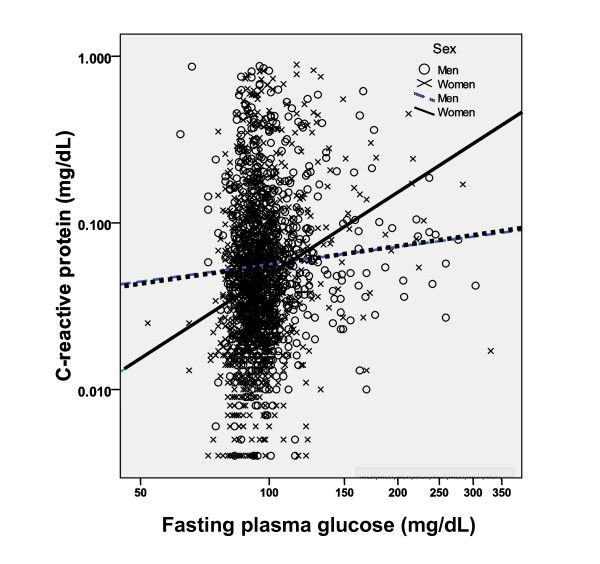
**Relationship between fasting plasma glucose (FPG) and high sensitivity C-reactive protein (hsCRP) according to gender**. In women, hsCRP increased significantly and progressively with increasing FPG (r = 0.169, *P *< 0.001). Test of significance was based on log-transformed values for analysis. *P *-value: Pearson's correlation coefficient.

In women only, hsCRP increased significantly and progressively with increasing FPG (r = 0.169, *P *< 0.001) (Figure 1).

### Geometric mean levels of hsCRP and standard error bar according to gender, obesity, and FPG category

**Figure 2:**
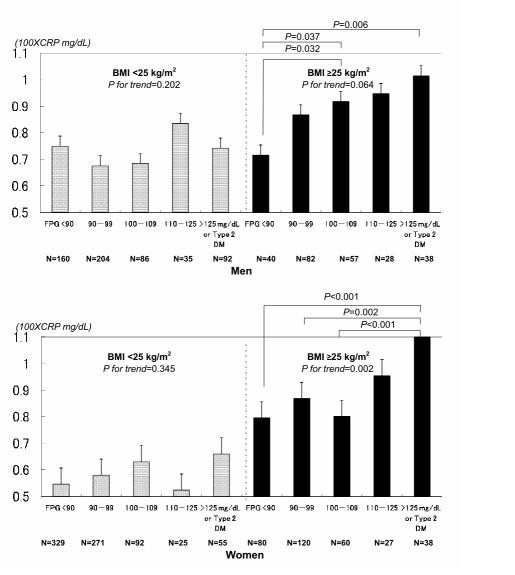
**Geometric mean levels of high sensitivity C-reactive protein (hsCRP) and standard error bars, according to obesity, and fasting plasma glucose (FPG) category**. Obesity was defined as a body mass index of ≥25 kg/m^2^. hsCRP levels were adjusted for age, smoking status, systolic blood pressure, high-density lipoprotein cholesterol, uric acid, and high molecular weight (HMW) adiponectin. Data for HMW adiponectin and hsCRP were skewed, and log-transformed for analysis.

Figure 2 shows the geometric mean levels of CRP (adjusted for age, smoking status, SBP, HDL-C, uric acid, and HMW adiponectin) according to gender, obesity, and FPG category. Adjusted geometric mean levels of hsCRP in obese (BMI ≥25 kg/m^2^) men with the lowest, second lowest, and highest values of normal FPG, IFG, and type 2 diabetes were 0.716, 0.867, 0.917, 0.947, and 1.014, respectively. The respective adjusted geometric mean levels of hsCRP for obese women were 0.796, 0.868, 0.801, 0.954, and 1.108, respectively (*P for trend *= 0.002). As shown, there was a significant gender interaction, and the increase in hsCRP levels was greater in women than men with obesity and a higher FPG Level.

## Discussion

In this cross-sectional, population-based study, we determined the relation between glucose levels and hsCRP by gender in healthy participants. This study showed that hsCRP levels increase continuously across the spectrum of FPG, starting from the lowest FPG in both men and women. When analyzing FPG and BMI simultaneously, it is apparent that increased hsCRP levels are mainly due to increased hsCRP concentrations according to the status of obesity. These findings suggest that the FPG-hsCRP relationship is stronger for subjects with obesity than for those without. In addition, increase in hsCRP levels was greater in women than men, and we demonstrated that there is a gender interaction between increased FPG and hsCRP after adjustment for age, BMI, smoking status, SBP, HDL-C, uric acid, FPG, and HMW adiponectin. To our knowledge, few studies indicate modification of the effect by gender in the association between FPG and inflammation in Japanese community-dwelling adults.

Several previous studies show that gender, FPG and obesity are independent confounding factors for increased hsCRP. In a total of 1,737 Korean subjects aged >60 years, multiple regression analysis using hsCRP as a dependent variable showed that BMI and white blood cell count as well as fasting insulin, post-load 2 h glucose, hematocrit and LDL-C were significant independent variables [16]. In 520 Chinese patients with type 2 diabetes, insulin resistance was associated with CRP levels independent of abdominal obesity [17]. Unek et al. [18] report that obese patients show a significant increase in hsCRP and soluble CD40 ligand levels compared with normal weight subjects, which might contribute to the known proinflammatory milieu found in these patients. In a total of 16,616 men and non-pregnant women aged ≥ 17 years, after adjustment for potential confounders, the adjusted OR of obese men and women for increased CRP was 2.13 (95% CI, 1.56-2.91) and 6.21 (95% CI, 4.94-7.81), respectively. In addition, BMI was associated with increased CRP levels in women only [19]. In a population-based cross-sectional study, fasting glucose remained significantly and independently related to CRP levels [10,11], and hsCRP as well as the insulin resistance indices increased gradually even in the normal fasting glucose range, supporting the rationale for expanding the range of fasting hyperglycemia [12]. Moreover, an increase in CRP levels was greater in women than men in the obesity and higher fasting glucose category, and this association was more pronounced in women [11]. In our study, increased FPG levels were positively associated with increased hsCRP, independent of other confounders, especially in obese women and the association was also shown to be within the normal range in obese participants.

There are several possible mechanisms linking FPG, gender, obesity and hsCRP. This result shows that high FPG levels can increase levels of key proteins (e.g., hsCRP) that result in inflammation. In experimental studies, human monocytes show a glucose-dependent increase in TNF-α and IL-6 production [20-22]. In female Turkish subjects, subjects with impaired glucose tolerance had higher fasting serum TNF-α levels than subjects with normal glucose tolerance (*p *< 0.01), and serum TNF-α and IL-6 concentrations were elevated during the glucose challenge (for each comparison, *p *< 0.01) [23]. It has also been demonstrated that IL-6 and high glucose synergistically stimulate matrix metalloproteinase-1 (MMP-1) expression in mononuclear phagocytes, and IL-6 and high glucose synergistically upregulate MMP-1 expression by U937 mononuclear phagocytes [24]. Obesity per se is also an important risk factor of the development of type 2 diabetes and is associated with low-grade systemic inflammation [25]. Furthermore, Fat accumulation in the liver or adipose tissues can induce inflammatory cytokines such as TNF-α, IL-6, and IL-8 [26]. These cytokines produced by adipocytes stimulate hepatic synthesis of CRP, which is an acute-phase protein, and influence insulin resistance as well as lipid and glucose metabolisms [3,27-29]. All these factors result in the production of CRP.

Other mechanisms may contribute to the altered glucose metabolism demonstrated. Adiponectin is an adipocyte-secreted cytokine, which has insulin sensitizing, anti-inflammatory, and anti-atherosclerotic properties [30,31], and is decreased in persons that are obese and have diabetes [32]. Decreased adiponectin could affect blood glucose and systemic inflammation. However, in our study, an association between FPG and hsCRP remained significant after adjusting for confounding factors including BMI and HMW adiponectin.

Our finding showed that gender interaction in the association between MetS and hsCRP suggest that hormones may play an important role in the inflammatory mechanism [33,34]. In our study, increased BMI was associated with increased hsCRP concentrations in both genders, however, the correlation coefficients between hsCRP and BMI were much higher in women (r = 0.355) than in men (r = 0.188). These results demonstrated that inflammatory response may be more enhanced through adipose tissue-secreted cytokine expression in women [11]. Thus, sex hormones may be responsible for the association of fasting glucose with hsCRP. In postmenopausal women, this gender-specific effect of fasting glucose on inflammation might help to explain an increase in the relative risk for MetS and CVD [35]

Several limitations should be considered in this study. First, our cross-sectional study design does not eliminate potential causal relationships between CRP and FPG. Second, FPG categories are based on a single assessment of blood, which may introduce a misclassification bias. Third, we could not eliminate the possible effect of medication for hypertension on the present findings. Therefore the demographics and referral source may limit generalizability.

In conclusion, the present study showed that FPG levels are strongly associated with hsCRP in the general population with obesity. The underlying mechanism behind this relationship is unclear, but seems to be independent of traditional cardiovascular risk factors such as age, BMI, smoking status, alcohol consumption, blood pressure, lipids, or uric acid. Thus, the FPG levels (especially, higher values of normal FPG, IFG, and women) in persons with obesity might provide an important marker for the assessment of atherogenic risk as well as a therapeutic target for the modification of atherogenic risks. For healthy community-dwelling persons, prospective population-based studies are needed to investigate the mechanisms underlying this association to determine whether intervention, such as effective lifestyle modifications and medication (e.g., antihypertensive, antilipidemic, and diabetic medication) that decrease BMI and FPG in adults, will decrease hsCRP.

## Competing interests

The authors declare that they have no competing interests.

## Authors' contributions

RK, YT, and KK participated in the design of the study, performed the statistical analysis and drafted the manuscript. NO, TK, and TK contributed to the acquisition of data and its interpretation. ST and MA contributed to the conception and design of the statistical analysis. TM conceived of the study, participated in its design, coordination and helped to draft the manuscript. All authors read and approved the manuscript.
